# Experience of transfer from child to adult mental health services of young people with autism spectrum disorder

**DOI:** 10.1192/bjo.2020.41

**Published:** 2020-06-03

**Authors:** Hannah Merrick, Chris King, Helen McConachie, Jeremy R. Parr, Ann Le Couteur

**Affiliations:** Population Health Sciences Institute, Newcastle University, UK; Northern Regional Child & Adolescent Psychiatry training scheme, UK; Population Health Sciences Institute, Newcastle University, UK; Population Health Sciences Institute, Newcastle University and Cumbria Northumberland, Tyne and Wear NHS Foundation Trust and Newcastle upon Tyne Hospital NHS Foundation Trust, UK; Population Health Sciences Institute, Newcastle University, UK

**Keywords:** Transition, autism spectrum disorder, mental health, adolescence

## Abstract

**Background:**

Transition from child-centred to adult mental health services has been reported as challenging for young people. It can be especially difficult for young people with autism spectrum disorder (ASD) as they manage the challenges of adolescence and navigate leaving child and adolescent mental health services (CAMHS).

**Aims:**

This study examines the predictors of transfer to adult mental health services, and using a qualitative analysis, explores the young people’s experiences of transition.

**Method:**

A UK sample of 118 young people aged 14–21 years, with ASD and additional mental health problems, recruited from four National Health Service trusts were followed up every 12 months over 3 years, as they were discharged from CAMHS. Measures of mental health and rich additional contextual information (clinical, family, social, educational) were used to capture their experiences. Regression and framework analyses were used.

**Results:**

Regression analysis showed having an attention-deficit hyperactivity disorder diagnosis and taking medication were predictors of transfer from child to adult mental health services. Several features of young people's transition experience were found to be associated with positive outcomes and ongoing problems, including family factors, education transitions and levels of engagement with services.

**Conclusions:**

The findings show the importance of monitoring and identifying those young people that might be particularly at risk of negative outcomes and crisis presentations. Although some young people were able to successfully manage their mental health following discharge from CAMHS, others reported levels of unmet need and negative experiences of transition.

## Background

For young people with ongoing mental health needs, and also their families, poorly planned transitions can make the shift from child and adolescent mental health services (CAMHS) to adult mental health services (AMHS) difficult.^1,2^ Transition refers to the purposeful, planned process that addresses the medical, psychosocial, educational and vocational needs of adolescents and young adults with chronic physical, neurodevelopmental and medical conditions as they move from child-centred to adult-oriented health-care systems.

Previous research has shown a third of young people experience a disruption or loss of care in the transition from CAMHS to AMHS, with only 4% experiencing an ‘ideal’ transition.^3^ Reasons for this include no longer needing clinical mental health involvement, presenting problems not meeting criteria for AMHS or disengagement from services.^4^

For some young people, discharge to primary care is appropriate. However, certain groups of young people such as those with emotional, neurodevelopmental (including autism spectrum disorder (ASD)), or emerging personality disorders are at particular risk of not accessing adult services and of ‘falling through the CAMHS–AMHS gap’.^3,5^

These young people may subsequently present to adult services following a crisis or develop more serious and enduring mental health problems.^6^ Transfer to AMHS (this refers to the formal event when medical care of a young person is moved from children's services to adults’ services) has been found to be predicted by being prescribed medication at the time of transition and having a severe or enduring mental health condition, such as schizophrenia, bipolar affective disorder or psychotic disorders.^3^

## Aims

This paper uses data from a UK longitudinal study of young people with long-term conditions (including ASD and additional mental health problems)^7^ to identify predictors of transfer to AMHS or discharge to primary care, and gain a greater understanding of the experience of transition for individual young people with both ASD and additional mental health problems. We hypothesised that (a) those young people who require medication, and/or had one or more severe mental health problem were more likely to transfer to AMHS; and (b) there would be evidence of unmet need for a substantial proportion of young people.

## Method

### Participants

Young people aged 14–18 years, with a diagnosis of ASD who were accessing CAMHS for an additional mental health problem, were recruited to the Transition Longitudinal research project (http://research.ncl.ac.uk/transition/) between 2012 and 2013. All were referred by clinicians who confirmed a diagnosis of ASD (through information in medical records), the additional mental health problem(s) and that the young people did not have a significant intellectual disability (also known in UK health services as a learning disability, confirmed by the referring clinician).

Young people were recruited from four services across England. Once consented, the families were visited on four occasions (baseline, 12-, 24- and 36-month follow-up) and completed several questionnaires. Full details of the study protocol and baseline characteristics have been published.^7^

The authors assert that all procedures contributing to this work comply with the ethical standards of the relevant national and institutional committees on human experimentation and with the Helsinki Declaration of 1975, as revised in 2008. All procedures involving human patients were approved by the Newcastle and North Tyneside 1 Research Ethics Committee (Numbers 12/NE/0059 and 12/NE/0284). All young people, and one parent/carer for each young person, provided informed written consent to join the study. young people under 16 years of age signed an assent form and the parent/carer gave informed consent.

### Measures

At baseline, the Social Responsiveness Scale (SRS)^8^ (completed by the parents) was used to indicate the extent of social impairment. The Strengths and Difficulties Questionnaire (SDQ:^9^ a measure of young people's emotional and behavioural problems) was completed independently by the young people and a parent.

The following measures were completed at each visit.

- A sociodemographic questionnaire: a bespoke questionnaire including information on gender, education and employment status and the Index of Multiple Deprivation (IMD) to assess socioeconomic deprivation.^10^
- The Hospital Anxiety and Depression Scale (HADS):^11^ a self-report 14-item questionnaire provided a measure of mental health symptoms for the week prior to the research visit. It has two subscales: anxiety and depression. Total scores categorised as ‘normal’ (0–7), ‘borderline abnormal’ (8–11) and ‘abnormal’ or clinical ‘caseness’ (12–21). An initial validation study has shown excellent psychometric properties in older adolescents and young adults with ASD.^12^
- The Warwick Edinburgh Mental Wellbeing Scale (WEMWBS):^13^ a 14-item, self-report questionnaire, developed in the UK and valid in the age range 13 to 21 years, captures young people's mental well-being.

### Information collated by the research assistants

Information was derived from clinical case notes and at research visits. Before each follow-up visit, the trained research assistants, with the young people's consent, accessed the clinical mental health case notes to record details of appointments (including whether the appointment was attended, prescriptions, diagnoses made and date of transfer); clinical information about the content of discussions between the clinician, young people and/or parent; issues raised by the young people or the parent in appointments/phone calls; and any referrals made to other support services.

At each face-to-face research visit, the questionnaires and notes from the clinical records were used to aid discussion with the young people and parent about their experience of healthcare services over the previous 12 months. A wide range of topics were covered including personal achievements and difficulties, family issues, changes in or problems at school or with the service provider, and access to or lack of relevant support. Following each visit, research assistants systematically collated this information about difficulties and complexities for the young person, their family and relevant professionals, together with positive successes and achievements.

### Data analysis

#### Quantitative data analysis

SPSS version 23 was used for data analysis. Selected variables were compared using ANOVA or χ^2^-tests, which informed the subsequent analysis. Logistic regression was used to identify predictors of transfer from CAMHS to either AMHS or discharge to primary care. To maximise the data available for analysis, data for the young people who completed three or four visits were included; ‘final visit’ data were created combining those who completed the 24-month follow-up and those who completed the 36-month follow-up visit.^14^ Out of the 118 young people recruited, 88 completed a final visit. There were no significant differences between those who remained in the study and those who withdrew from the study in terms of condition severity or sociodemographic factors.^14^ Missing-item level data were handled according to the recommended rules for each outcome measure.

#### Analysis of information collated by research assistants

**Fig. 1:**
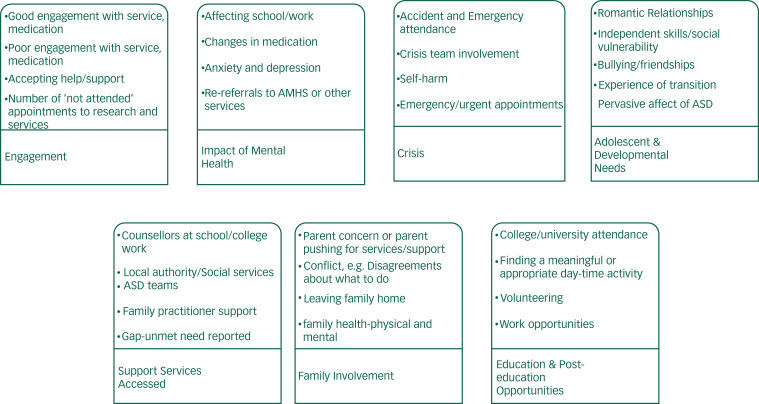
Categories from framework analysis.

AMHS, adult mental health services; ASD, autism spectrum disorder.

Framework analysis^15^ was used to consider and organise these data. H.M and C.K. repeatedly read through the information, identifying initial emerging themes and developed a thematic framework to facilitate the exploration of patterns and associations in the data. A 10% sample was double-coded to ensure consistency and reliability. The themes were then refined into categories (Fig. 1). Any differences were discussed and resolved through consensus. The themes and categories were reviewed and finalised with A.LeC.).

#### Mental health trajectories

The trajectories of the young people's HADS scores over the 3-year study period were reviewed. Of the 88 young people who completed a final visit, 82 had sufficient complete HADS data. Using the sequence of self-reported HADS scores, participants were grouped into three trajectory types:

- ‘doing well’: ‘normal’ HADS scores across all visits or showing improvement from abnormal or borderline abnormal scores to normal scores at the final visit;
- ‘continued moderate difficulty’*:* borderline abnormal mental health problems over the visits or scores fluctuating between borderline and abnormal; and
- ‘not doing well’: continued abnormal HADS scores or a decrease in scores over the study period ending in the abnormal range.

The trajectory of each young people was independently assessed by H.M. and C.K. and a consensus agreed.

## Results

### Participant characteristics

In total, 118 young people (mean age 16.1 years, s.d. = 1.3, range 14–18.9) with ASD and additional mental health problems and 113 parents/carers completed baseline measures (69.5% men, 30.5% women); 98.3% described themselves as White British. The mean age of young people at the final visit was 19.1 years (s.d. = 1.4, range: 16.1–21.9 years). Therefore, although the young people were of a chronological age to be discharged from children's services, the sample was still relatively young in terms of transition, which can be expected to continue up to 25 years of age.

At baseline young people-reported and parent-reported SDQ scores were borderline abnormal and abnormal, respectively (mean 17.6, s.d. = 6.1; mean 22.8, s.d. = 5.9, respectively).^9^ SRS scores were above the cut-offs for screening of ASD for both male (>70; mean 120, s.d. = 29.4) and female (>65; mean 110.4, s.d. = 29.6) participants.^8^ WEMWBS scores at baseline (median 47, interquartile range (IQR) = 41–52) were significantly below population norms.^16^ At the final visit 88 young people remained in the study, giving a retention rate of 74.6%.

### Transfer location

Thirty young people withdrew from the study. Transfer location information was available for five of these, so their data were included in the analyses. Transfer data for 93 young people are presented in Table 1.

**Table 1:** Characteristics of sample by transfer location

	Remained in:	Transferred to…^a^				
	CAMHS^b^	Primary care	Adult services	ANOVA, *F* (d.f.)	χ^2^ (d.f.)	*P*
Total, *n*	20	48	25	–	–	–
Gender, *n* (%)						
Male	16 (80)	31 (65)	16 (64)	–	–	–
Female	4 (20)	17 (35)	9 (36)	–	–	–
Age at visit before transfer						
Mean (s.d.)	17.7 (0.89)	17.4 (0.89)	18.2 (0.69)	6.97 (2, 90)		<0.05
Range	16.13–20.30	14.7–19.75	16.5–19.9			
WEMWBS^c^						
Mean (s.d.)	48.4 (8.80)	45.6 (7.99)	46.52 (10.17)	0.74 (2, 90)		0.48
Range	28–58	29–60	27–70			
IMD at baseline						
Mean (s.d.)	18.98 (11.36)	23.94 (18.13)	24.45 (18.74)	0.72 ((2, 90)		0.49
Range	2.46–38.82	2.17–80.51	3.72–71.83	–		
Prescribed medication	16 (80)	27 (56)	20 (80)		5.995 (2)	0.05
Education and employment						
Full/part-time education	15 (75)	41 (85.4)	21 (84)			
Employed	4 (20)	1 (2)	2 (8)			
Not in education or employment	1 (5)	6 (12.5)	2 (8)			
Number of mental health problems						
1	8 (40)	27 (56)	15 (60)			
≥2	12 (60)	16 (33)	10 (40)			
Mental health problem^a^						
ADHD/ADD	12 (60)	8 (16.7)	15 (60)		18.582 (2)	<0.05
Mood^d^	2 (10)	18 (38)	7 (28)		5.200 (2)	0.07
Anxiety^e^	9 (45)	22 (46)	6 (24)		3.560 (2)	0.169
ODD/challenging behaviour^f^	2 (10)	5 (10)	1 (4)			
Sleep disorders^g^	6 (30)	7 (15)	4 (16)			
Other^h^	4 (20)	1 (2)	3 (12)			
Self-harm	1 (5)	2 (4)	5 (20)			
HADS score (anxiety)						
<8	7 (35)	20 (42)	10 (40)		0.263 (2)	0.88
≥8	13 (65)	28 (58)	15 (60)			
HADS score (depression)						
<8	18 (90)	39 (81)	17 (68)		3.480 (2)	0.18
≥8	2 (10)	9 (19)	8 (32)			
Presence of developmental disorder^i^	3 (15)	5 (10)	5 (20)			
Presence of physical health problem^j^	11 (55)	12 (25)	10 (40)			

CAMHS, Child and adolescent mental health services; WEMWBS, Warwick Edinburgh Mental Wellbeing Scale; IMD, Index of Multiple Deprivation; ADHD, Attention deficit hyperactivity disorder; ADD, attention-deficit disorder; ODD, oppositional defiant disorder; HADS, Hospital Anxiety and Depression Scale.

a. Young person transferred over the 3-year study period. Data is from the visit after their transfer.

b. Young person was still in CAMHS at final visit. Data are from final visit.

c. Significantly below population norms across all groups. χ^2^ analysis not conducted for some conditions because of the low frequency of occurrence.

d. Depression, low mood.

e. Anxiety, obsessive–compulsive disorder, phobia, social anxiety.

f. Oppositional defiant disorder, conduct disorder, challenging behaviour, behavioural problems, aggression, anger management problems.

g. Insomnia, requiring melatonin.

h. Conversion disorder, psychosis (one young person), chronic fatigue syndrome.

i. Dyslexia, dyspraxia, dyscalculia, developmental coordination disorder.

j. Asthma, epilepsy (one young person), allergies, migraine, thyroid dysfunction.

There were differences in the ages of each transfer group, with overlap between groups. This is not surprising given that some young people were discharged to primary care earlier than they would be transferred to AMHS. Building on these findings a stepwise logistic regression was used to identify predictors of transfer from CAMHS to either AMHS or discharge to primary care. Taking prescribed medication was entered first, followed by a diagnosis of attention-deficit hyperactivity disorder (ADHD), and the remaining variables. Two individuals from the primary care group were not included in the analysis as they were aged under 16 years when discharged from CAMHS and therefore not eligible to transfer to AMHS.

The logistic regression findings are presented in Table 2. Overall the final model that included a diagnosis of ADHD (odds ratio (OR) = 8.22, 95% CI 2.33–29.02, *P* = 0.001) and prescribed medication for a mental health problem (OR = 4.00, 95% CI 1.00–15.95, *P* = 0.05), was significant in predicting transfer outcome (χ^2^ (7) = 18.58, *P* = 0.010). Nagelkerke *R*^2^ indicates that the final model explains 31.7% of the variance. Prescribed medication was only significant with the introduction of an ADHD diagnosis in the model; therefore, this significance is likely to be explained by young people with ADHD accessing adult services for medication.

**Table 2:** Logistic regression for predictors of transfer location

Variable^a^	*n* (%)	Block 1	Block 2	Block 3
Nagelkerke *R*^2^, %	–	6.5	30.6	31.7
Prescribed medication, OR (95% CI)				
Yes	47 (66)	2.82 (0.90–8.82)	3.85 (1.03–14.41)*	4.00 (1.00–15.95)*
No	24 (34)			
Additional mental health problem, OR (95% CI)				
ADHD	23 (32)	–	8.59 (2.60–28.43)*	8.22 (2.33–29.02)*
Not ADHD	48 (68)	–		
Number of mental health problems, OR (95% CI)				
1	45 (63)	–	–	1.04 (0.31–3.43)
≥2	26 (37)	–	–	
HADS Depression, OR (95% CI)	–	–	–	1.03 (0.85–1.26)
HADS Anxiety, OR (95% CI)	–	–	–	1.06 (0.89–1.25)
WEMWBS, OR (95% CI)	–	–	–	0.99 (0.96–1.03)
IMD score (baseline), OR (95% CI)	–	–	–	1.03 (0.94–1.14)

OR, odds ratios; ADHD, Attention-deficit hyperactivity disorder; HADS, Hospital Anxiety and Depression Scale; WEMWBS, Warwick Edinburgh Mental Wellbeing Scale; IMD, Index of Multiple Deprivation.

a. Data are from the visit following the young person's transfer date, except for IMD, which was collected at baseline.

* Significant at *P* < 0.05.

### Analysis of additional contextual data

The framework analysis used data from the total sample (*n* = 118). Twenty-four themes were identified and then summarised into seven categories that described the young people's and their parents’ experience of transition (see Fig. 1).

Several of the themes identified related to concerns about ASD and broader developmental and adolescent issues.

### Mental health trajectory

Young people were grouped into three HADS trajectories: ‘doing well’ (*n* = 23), ‘continued moderate difficulty’ (*n* = 29), ‘not doing well’ (*n* = 30). There were no significant differences in transfer outcome, age, baseline SRS scores, or additional mental health diagnoses between the groups (Table 3).

**Table 3:** Differences between Hospital Anxiety and Depression Scale trajectory groups

	‘Doing well’, *n* = 23	‘Moderate difficulty’, *n* = 29	‘Not doing well’, *n* = 30	ANOVA, *F* (d.f.)	χ^2^ (d.f.)	*P*
CAMHS, *n* (%)	6 (26.1)	5 (17.2)	7 (23.3)	–	–	–
Transfer location, *n* (%)						
Primary care	10 (43.5)	15 (51.7)	16 (53.3)	–	0.53 (2)	0.767
AMHS	7 (30.4)	9 (31)	7 (23.3)		–	–
Gender, *n* (%)						
Female	3 (13)	9 (31)	18 (60)	–	12.97 (2)	0.002
Male	20 (87)	20 (69)	12 (40)		–	–
Young Person SDQ, Total:^a^ mean (s.d.)	14.17 (3.64)	19.49 (4.46)	20.38 (6.67)	10.31 (2,77)	–	<0.001
Parent SDQ Total,^a^ mean (s.d.)	22.09 (5.25	22.45 (5.28)	22.23 (6.89)	0.02 (2,74)	–	0.977
SRS Scores,^a^ mean (s.d.)	114.41 (19.74)	123.41 (27.92)	109.38 (31.78)	1.85 (2.77)	–	0.164
WEMWBS,^b^ mean (s.d.)						
Visit 1	49.96 (7.78)	44.0 (8.24)	42.57 (8.95)	16.13 (2,72)	–	<0.001
Visit 2	52.27 (6.94)	45.25 (8.41)	41.89 (9.13)		–	–
Final visit	53.96 (8.08)	46.61 (7.57)	41.32 (10.32)		–	–

CAMHS, Child and adolescent mental health services; AMHS, Adult mental health services; SDQ, Strengths and Difficulties Questionnaire; SRS, Social Responsiveness Scale; WEMWBS, Warwick Edinburgh Mental Wellbeing Scale.

a. Higher scores reflect more difficulties on the SDQ and greater social impairment on the SRS.

b. Higher scores reflect better mental well-being.

There were significantly more female young people in the ‘not doing well’ group compared with the ‘doing well’ group (χ^2^(1) = 11.99, *P* = 0.001) and the ‘moderate difficulty’ group (χ^2^(1) = 4.99, *P* = 0.026). Those in the ‘doing well’ group reported significantly lower baseline young person-reported SDQ scores than the ‘moderate difficulty’ (*P* = 0.001) and the ‘not doing well’ groups (*P* < 0.001). There was no difference in young person-reported SDQ scores between the ‘moderate difficulty’ group and the ‘not doing well’ group (*P* = 0.792). Across all the time points those in the ‘doing well’ group reported significantly higher well-being (WEMWBS) scores than the ‘moderate difficulty’ (*P* = 0.001) and the ‘not doing well’ groups (*P* < 0.001). There was no significant difference between the ‘not doing well’ and the ‘moderate difficulty’ group (*P* = 0.154).

The source materials used to identify the categories and themes identified in the framework analysis (see Fig. 1) also enabled an exploration of aspects of transition and mental health experiences relevant for individuals within the HADS groups. A summary and examples of differences between the ‘doing well’, ‘continued moderate difficulty’ and ‘not doing well’ groups are shown in the Appendix.

Those in the ‘doing well’ group had made positive references to levels of engagement with services and there were accounts of relative stability in terms of education achievements and family life compared with the other two groups. Results suggest that individuals in this group were learning to manage their mental health concerns, developing an awareness of the impact of their ASD and learning to negotiate, with support, some developmentally appropriate aspects of transition to adulthood.

In the ‘moderate difficulty’ group, the young people reported life events such as family illness, parental separation or disrupted education transitions that may have contributed to fluctuations in their self-reported mental health. There were also reported concerns about the effect of ASD characteristics on independence. This group seemed to be experiencing difficulties in one or two areas, whereas in the ‘not doing well’ group, there seemed to be difficulties across multiple areas. Similar to the ‘moderate difficulty’ group, the research visit notes reported the adverse impact of ASD symptoms (such as limited independent living skills, problems in maintaining friendships), and the families’ and professionals’ awareness of unmet needs. However, for many, longer-term disengagement meant young people were not accessing the services and support offered to them, despite appearing to have a greater need.

It is not possible to draw conclusions about causality in relation to whether fewer ASD-related and family/social issues resulted in better engagement and mental health outcomes for the ‘doing well’ group, and vice versa for the ‘not doing well’ group. The presence of multiple difficulties and challenges in all these areas appeared to be associated with negative outcomes for young people in the ‘not doing well’ group. In the ‘not doing well’ group, young people appeared often to be being referred to and have contact with several different National Health Service and volunteer organisations. However, these contacts were often short-term either because of the nature of the referral or to disengagement. Potentially, having these short-term contacts with several different organisations was a struggle for this group to manage and feel supported in, especially given their greater self-reported SDQ scores.

## Discussion

### Main findings

For young people with ASD and additional mental health problems in our study, despite having persisting symptoms of anxiety and depression, the only significant predictors of transfer from CAMHS to AMHS were having a diagnosis of ADHD and taking prescribed medication (when combined with a diagnosis of ADHD). The number of mental health disorders, HADS scores and well-being scores did not predict adult healthcare outcome.

Our findings from the regression analysis are in contrast to previous research that highlighted the problems for young people with ASD and ADHD achieving successful transfer to AMHS.^3,17^ The UK ADHD National Institute for Health and Care Excellence guidelines^18^ NG87 recommend that both diagnosis of ADHD and the initiation of drug treatment should be undertaken by appropriately qualified healthcare professionals with expertise in ADHD, and that continued prescribing and monitoring of drug therapy may be performed by primary care physicians, under shared care arrangement with the specialist services. No reference is made in NG87 about individuals with co-occurring ASD. It is unclear whether UK AMHS are adequately resourced to provide diagnostic and follow-up clinics for adults with ASD, ADHD and other additional co-occurring mental health conditions. Our findings might imply that for individuals with ASD, the ADHD symptoms may be particularly impairing and so lead to transfer to AMHS. Indeed, several parents and young people commented that 6 monthly/yearly follow-up medication clinic appointments did not meet the wider needs of young people coping with a broad range of concerns about mental health and other social and educational needs.

### Transfer outcomes

Across the three HADS trajectory groups there was no significant difference in their transfer outcome, despite one group having continuous or increasing difficulty with their mental health. The qualitative results provide more detail about the experience of transition for young people with ASD and additional mental health problems. Not all young people with mental health problems either want or need to transfer to AMHS, with discharge to primary care being an appropriate pathway for some. Much has been written about the variations in eligibility-based adult service provision and the limited access to AMHS for young people with a range of mental health problems (including ASD and additional mental health problems, emotional disorders and emerging personality disorders).^3,5^ This may mean that at age 18 years some young people with ASD may find that, despite no change in their mental health problems, they can no longer access secondary mental health services.

For 35% of young people discharged to primary care, this pathway was not successful, resulting in crisis team involvement, referrals to counselling services, and receiving time-limited support in AMHS before being discharged again. The analysis of the HADS trajectories illustrated that some young people appeared to be more resilient, for example, managing their mental health and able to engage successfully with services. As has been found in previous research,^4^ skills such as young people's engagement with services/treatment seem to have an important role in successful transition, with some young people reportedly being discharged from CAMHS because of disengagement with services.

### Engagement

In our study, the most positive comments regarding engagement were from the ‘doing well’ group. This group did not differ significantly from the other groups in terms of severity of autism characteristics (baseline SRS scores), transfer outcome, age, additional mental health diagnoses or parental SDQ scores. However, the young people reported lower SDQ scores, higher well-being and consistently had either low (‘normal’) or reported improvement in HADS scores over the 3 years. These young people, their families and clinicians described constructive interactions about wider transition topics and aspects of ASD as well as management of mental health comorbidities. The analysis of the available contextual information from clinical and research notes for all groups highlighted the relevance of some of the young people’s broader ASD social and emotional developmental needs and adaptive learning skills.

Consistent with previous research, positive influences on the young people’s experience of transition seem to include gaining an ability to participate socially, engage consistently and constructively with services, positive support including employment opportunities from family, and access to other community resources including education, all of which may be more challenging for young people with ASD.^19,20^

### Transition contexts

Our analysis of HADS trajectories also highlighted a gender difference, with significantly fewer female participants in the ‘doing well’ group and significantly more in the ‘not doing well’ group. Although there is an increasing literature on the specific experiences of able women with ASD, few studies have specifically reported the transition experiences of young women with ASD. Perhaps for able young women the focus on social relational expectations and pressures of peer relationships with other young people may pose additional pressures during adolescence and transition – a period of considerable uncertainty. In a mixed methods study of able adolescents with autism and neurotypical adolescents, it was identified that the adolescent girls with autism had similar types of friendships and social experiences as their neurotypical peers, but had a smaller number of ‘intense’ friendships rather than a wider friendship group.^21^ The adolescent girls with autism experienced more conflict and relational victimhood (for example exclusion, manipulation, victim of rumours), and found conflict harder to manage successfully. They reported that friends, although a useful source of social support, ‘were hard work’ and that maintaining more than one or two friendships was difficult. This in turn meant that arguments could be devastating as ‘you then have no-one else to go to’.

Although their study did not consider the impact of transition, the findings highlight the importance of considering the potential consequences of relational aggression on mental health and well-being, perhaps especially for girls with autism. In contrast, a qualitative study identified a group of young people with ASD seen in childhood and interviewed them 12 years later.^22^ The thematic analysis identified that the young people (at age 16–20 years) felt more in control of their own lives, needed to take one step at a time and valued their ‘social connections with others’. Both these studies highlight the importance of the wider context for the young person, and that acknowledging the young people's need for more time to take on young adult roles and responsibilities, is likely to promote well-being and a sense of self-identity. Minimising the impact of disruption of mental health provision or loss of support also appeared in this study to promote continuation of underlying skills development and the ability of young people with ASD to make and maintain relationships.

### Identifying those most at risk of negative outcomes

How should we identify those young people that might be particularly at risk of negative outcomes through the period of transition? This study suggests that engaging with young people, and using a regular self-report check such as the HADS may help young people, their families and the professionals supporting them to identify their own trajectory and the impact of individual and family life experiences. This led to discussion of how to manage mental health needs or ASD-related needs that may be hindering their personal goals, achievements and engagement with support services. There is also a need for greater understanding of why some young people are disengaging from services.

The reasons varied within this study – for example, some young people chose not to be referred to or engage with AMHS because of reduced symptoms/impairment and use of effective coping strategies. Other young people underestimated their symptoms, disliking a dependence on therapies/medication and a rejection of support services, potentially leading to crisis and re-referral. Taking an approach that plans for the cessation of services or young people’s disengagement from services may result in better outcomes than unplanned disengagement.^23^

CAMHS services should consider teaching young people to recognise their own symptoms and impairments, and triggers for crises, to manage their mental health, and to participate in shared decision-making; this may improve outcomes following a gap or cessation of CAMHS/AMHS input. This may be especially important for those young people identified as at risk of disengagement. In a similar vein, it has been suggested that services should have easier re-entry policies (for example an open door policy) to help young people manage their mental health.^23,24^ Certainly, in this study, young people in the ‘not doing well’ group, reported struggles of trying to re-engage with AMHS when difficulties occurred following discharge from CAMHS or because of disengagement, leaving them with feelings of unmet need.

### Unmet needs

A common concern raised by parents and young people (especially for those young people with more abnormal HADS scores or with a deteriorating trajectory of scores) was their perceived lack of support and ‘unmet need’ regardless of whether the young people had been discharged to primary care or AMHS. These identified unmet needs reflect findings from other research where young people in the average range of intellectual ability with ASD can fall short of criteria for access to community intellectual disability or more specialist ASD services as well as community mental health services.^25,26^ The findings from this study highlight the relevance of current guidelines to improve holistic support for young people and adults with ASD.^27^ Access to local community expertise around interpersonal support, advice and information, especially if the expertise includes working with young adults with ASD and their families to identify the young people’s particular strengths, goals, skills and needs, could help increase participation and reduce this unmet need.^28^ Understanding the broader contexts of the young person, possibly beyond the scope of the appointment they are (or are not) attending and the factors that may be making engagement in medication, treatment and clinic appointments more challenging may highlight ways in which they need more support, or areas of priority for the young person at that time.

### Strengths and limitations

The sample size is comparable with similar studies looking at mental health transition and is one of the largest for ASD transition research.^3,19^ This study provides new insights in relation to young people's mental health and experiences over a crucial 3-year period during which the young people transferred from CAMHS. It is a secondary analysis of data collected during the Transition Longitudinal study.^28^ Although we were not able to undertake in-depth interviews with this relatively large number of young people and parents, we were able to collate accounts from all participants (young people and parents) over a 3-year period using clinical notes and systematically collected information from the annual face-to-face follow-up visits. The comprehensive and consistently collected data has allowed an opportunity to gain some insight into these young people's lived experience of transition. The young people are a relatively young sample (aged 14–17 years at recruitment; 17–21 at final visit). Thus, although all young people were approaching or had experience of planning for or achieving transfer from CAMHS, 20 young people had not left CAMHS by the end of the study (including 4 participants who were over the age of 18 years). Further, we do not have follow-up information for any individuals over 21 years.

### Implications

In conclusion, discharge from CAMHS could be seen as a new beginning for young people with ASD: moving on to further education opportunities; gaining an understanding about how to manage their mental health difficulties and acquiring adaptive life skills to address their developmental needs. However for others, ongoing mental health difficulties, social, emotional and relationship needs (particularly associated with ASD), and a feeling of lack of understanding and a perceived absence of professional understanding about their and their families’ level of unmet need with regard to both mental health and local authority services, contributed to a negative experience of transition and access to health and social care services.

In this study, a relatively small number of individuals had multiple negative experiences of service provision, struggled with engagement, felt unsupported, and presented with multiple crises over the study period. We propose that the use of a tool such as the HADS may be a useful adjunct for individuals and their supporting clinicians to identify patterns of mental health functioning over time; the monitoring may help identify those young people especially at risk of negative outcomes and crisis presentations. Successful transfer to AMHS is only one aspect of mental health support for some young people. However, whatever the adult healthcare provision all need access to expertise in ASD.

This study confirms the need to increase community practitioner clinical skills relevant to young people with ASD and additional mental health problems and highlights a need to take a holistic, person-centred approach to investigate whether or not there are any particular vulnerabilities for young people with mental health problems and without significant intellectual disabilities as they negotiate transition. The healthcare transition is one of many transitions young people with autism and mental health problems will be making, and they may be facing difficulties in their transition from education to employment, and finding appropriate goal-setting support.^29^

In particular, understanding families’ vulnerabilities is important for their successful transition. Our findings strongly support the potential benefit of a more nuanced approach to identifying and prioritising the needs of those young people at greatest risk of poor outcomes.^20^ Clinicians and other professionals with specialist expertise from children's and adult services could then support these young people and their families identify and prioritise their goals for timely community support before they are discharged from CAMHS.

## Data Availability

Authors have access to the original study data.

## Appendix

**Appendix:** Examples of themes and responses from the three Hospital Anxiety and Depression Scale trajectory groups

	‘Doing well’	‘Moderate difficulty’	‘Not doing well’
Engagement	Young people had few ‘Did not attend’ and were engaged well with therapies and medication regimes. ‘[Young person] reported that he uses cognitive behavioural therapy techniques on his own to manage his condition/anxiety/OCD [obsessive–compulsive disorder] symptoms when needed.’ (clinical notes)	Mixed group with some engaging well with services but also an increased reference to unattended appointments, sometimes leading to discharge. ‘Discharged as they cannot offer the young person anymore if he is not willing to try new strategies.’ (clinical notes)	Accounts of poor attendance and compliance at health service appointments. Details of young people refusing treatment options or support. ‘Young person left clinic due to appointments running late. Later called asking for prescription but got aggressive and hung up after being told they should still have 10 days left.’ (clinical notes)
Impact of mental health	Mental health problems were being successfully managed or had significantly declined. Young people were employing strategies learned to manage their mental health. When problems did arise, they were being referred to and engaging in an appropriate clinic. ‘Patient is no longer taking medication and is doing fine without it. Went to ask to be put back on ADHD [attention-deficit hyperactivity disorder) medication to help with concentration at college. Referral Adult ADHD services. He did not access adult services for his ADHD. He is using exercise and yoga to help manage his anger.’ (research visit notes)	Within this group changes in medication (either increases or reductions) were described because of fluctuations in mental health. Often periods of increased anxiety or depression described because of changes in school and family life. ‘Young person was very low in mood this visit – completely different from how he was for the first visit. Very subdued and apparently has had a few meltdowns recently. Medication gradually increased.’ (research visit notes)	Appeared that their autism spectrum disorder (ASD) was having a considerable adverse impact on the young person's progress. There were several comments in the clinical records of the young person being given details of ASD teams to access additional support, for example, to access services/support for training in friendships, socialising and independence skills. ‘Given literature from NAS [UK National Autistic Society] and list of books specific to ASD. Referral to Social Eyes service designed to help people with ASD to develop their social understanding and social skills.’ (clinical notes)
Crisis	Very few reports of self-harm or emergency situations.	Occurrences of need for urgent appointments and events of self-harm. ‘Young person struggling with panic attacks at university, low mood and some thoughts of suicide therefore urgent appointment given.’ (clinical notes)	Accounts describing episodes of self-harm, overdoses, suicide attempts and police involvement. Involvement of crisis intervention teams and some had also experienced in-patient care stays. ‘Between clinical appointments there were several calls between mum and psychiatrist and ICTS [intensive community treatment service] team when young person was “out of control”, with threats of self-harm. Mum advised to call police and called ICTS. Mum concerned about young person's behaviour and wants him hospitalised. Psychiatrist spoke to ICTS but they could not help as it is a longstanding issue.’ (summary from clinic notes)

Adolescent and developmental needs	Evidence that clinicians were considering the broader developmentally appropriate tasks and needs, such as transitioning from school to college, involvement in romantic relationships, encouraging increases in independence in appointments, and young person's awareness about how ASD may be affecting them. ‘Clinician and young person discussed…current romantic relationship: “emotional distress tends to be focussed around his girlfriend, who was also a close family friend” - talked about how his Asperger's might affect his relationship’ (clinical notes)	Descriptions of young people engaging in social activities but also more reports from parents of wanting more support for the young person in engaging in social activities, work and independent living. Some cases of safeguarding issues being raised, for example with the young person's internet use. ‘Young person recently joined an art group and singing group to help improve self-esteem and confidence. Futures team has helped young person fill in a transition plan that she also has a copy of. Mum is still very concerned about young person's lack of independent and social skills.’ (research visit notes)	Descriptions of young people and parents wanting more support with skills for independence and socialising. Accounts of discussions with clinicians about drug use and other risky health behaviours. ‘Educational advice given regarding physical and psychological effects of legal highs. Asked young person to complete summary of positive supports she has towards reducing drug use. Discussed importance of cooking three healthy meals a day for herself. Also discussed the importance of using contraception, especially in relation to her other medication.’ (clinical notes)
Support services accessed	Young people were accessing and using a range of support services including school counsellors, autism information services, social services and career services. Comments of agreeing with discharge to primary care for continued care. ‘Young person reported managing okay with just having a Social Services key worker helping him instead and a college counsellor. Young person opted not to be referred to AMHS as he was told he didn't meet the eligibility criteria for adult services.’ (research visit notes)	While some were engaging with additional services such as school counsellors and IAPT services, there were also comments about families feeling unsupported as a consequence of discharge or infrequent appointments. ‘Father reported they only see someone once every 6 months or so now, which he doesn't feel is enough. Father said he especially would like more help for his son with transitioning to adulthood and support with work etc. Said they currently see a different doctor every time they go so feel it is very impersonal.’ (research visit notes)	Some participants were accessing specialist services such as drug and alcohol support services or anger management teams following referrals from child and adolescent mental health services (CAMHS)/adult mental health services (AMHS). Accessing similar additional counselling and autism information services but described by the families as being less helpful or supportive. ‘Details given for Counselling Service. Assessed for input from Community Asperger's support team but young person did not meet criteria. Young person does not feel like her social worker is helpful in any way.’ (clinic notes)
Family involvement	Few reports of disrupted family life. Parents involved in appointments and supportive of young person's treatment decisions. Some discussion of other siblings/family members who also have mental health problems.	Young people reported life events such as family illness and parental separation that may have contributed to fluctuations in their self-reported mental health. ‘Mother and the young person were involved in a car accident recently. Mother been signed off work. FISS [family intensive support service] involvement in the family to think about how CAMHS can offer systemic support to the family. Discussed ongoing concerns within family and ongoing distress.’ (clinical notes)	Descriptions of parent's own mental health problems. Accounts of disagreements between parent and young people about treatment. ‘Art therapist discussed with young person difficulties with both parents also having input from mental health services.’ (clinic notes)
Education and post-education opportunities	Many of the young people were attending college or school and doing well. Some had plans to go to university or were looking at career options. Descriptions of young people gaining some part-time employment or volunteer work placements. ‘He is doing well with his job car painting and is hoping he will have a job at the end of his apprenticeship.’ (research visit notes)	Education experiences of the young people were mixed with some attending special needs schools, or dealing with disruptions, for example, being out of education for some time because of bullying or exclusion or the challenges around post-school transition, such as difficulties settling in and accessing available support at further education/few university. ‘[Young person] is now at university. Mum said [Young person] had a few panic attacks to begin with but is doing ok. [Young person] has a counsellor at university.’ (research visit notes)	Case notes detailed problems finding appropriate post-school placements and time out from education. Encouragingly, a couple of the young people had been able to obtain full-time work with the help of family members following dropping out of education. ‘[Young person] was expelled from college so waiting to see if he can go back in the near future or September. He is now working full time with his dad.’ (research visit notes)

## References

[1] Blum RW, Garell D, Hodgman CH, Jorissen TW, Okinow NA, Orr DP, Transition from child-centered to adult health-care systems for adolescents with chronic conditions: a position paper of the Society for Adolescent Medicine. J Adolesc Health 1993; 14: 570–6. 8312295 10.1016/1054-139x(93)90143-d

[2] Saqr Y, Braun E, Porter K, Barnette D, Hanks C. Addressing medical needs of adolescents and adults with autism spectrum disorders in a primary care setting. Autism 2017; 22: 51–61. 28750547 10.1177/1362361317709970 PMC5788079

[3] Singh SP, Paul M, Ford T, Kramer T, Weaver T, McLaren S, Process, outcome and experience of transition from child to adult mental healthcare: multiperspective study. Br J Psychiatry 2010; 197: 305–12. 20884954 10.1192/bjp.bp.109.075135

[4] Islam Z, Ford T, Kramer T, Paul M, Parsons H, Harley K, Mind how you cross the gap! Outcomes for young people who failed to make the transition from child to adult services: the TRACK study. BJPsych Bull 2016; 40: 142–8. 27280035 10.1192/pb.bp.115.050690 PMC4887732

[5] Paul M, Ford T, Kramer T, Islam Z, Harley K, Singh SP. Transfers and transitions between child and adult mental health services. Br J Psychiatry Suppl 2013; **202** (suppl 54): s36–40. 23288500 10.1192/bjp.bp.112.119198

[6] National Institute for Health and Care Excellence. Transition from Children's to Adults’ Services for Young People using Health or Social Care Services. NICE, 2016.

[7] Colver A, Rapley T, Parr JR, McConachie H, Dovey-Pearce G, Le Couteur A, Facilitating the transition of young people with long-term conditions through health services from childhood to adulthood: the Transition research programme. NIHR Journals Library, 2019. 31116547

[8] Constantino J, Gruber C. Social Responsiveness Scale. Western Psychological Services, 2005.

[9] Goodman R, Meltzer H, Bailey V. The Strengths and Difficulties Questionnaire: a pilot study on the validity of the self-report version. Eur Child Adolesc Psychiatry 1998; 7: 125–30. 9826298 10.1007/s007870050057

[10] Ministry of Housing, Communities & Local Government. *English IMD 2010 Data* Ministry of Housing, Communities & Local Government, 2011 (https://www.gov.uk/government/statistics/english-indices-of-deprivation-2010).

[11] Zigmond AS, Snaith RP. The hospital anxiety and depression scale. Acta Psychiatr Scand 1983; 67: 361–70. 6880820 10.1111/j.1600-0447.1983.tb09716.x

[12] Uljarević M, Richdale AL, McConachie H, Hedley D, Cai RY, Merrick H, The hospital anxiety and depression scale: factor structure and psychometric properties in older adolescents and young adults with autism spectrum disorder. Autism Res 2018; 1 **1**: 258–69. 10.1002/aur.1872 28922575

[13] Clarke A, Friede T, Putz R, Ashdown J, Martin S, Blake A, Warwick-Edinburgh Mental Well-being Scale (WEMWBS): validated for teenage school students in England and Scotland. A mixed methods assessment. BMC Public Health 2011; 11: 487. 21693055 10.1186/1471-2458-11-487 PMC3141456

[14] Colver AF, McConachie H, Le Couteur A, Dovey-Pearce G, Mann KD, McDonagh JE, A longitudinal, observational study of the features of transitional healthcare associated with better outcomes for young people with long-term conditions. BMC Med 2018; 16: 111. 30032726 10.1186/s12916-018-1102-y PMC6055340

[15] Ritchie J, Spencer L, O'Connor W. Carrying out qualitative analysis In Qualitative Research Practice: A Guide for Social Science Students and Researchers (eds J Ritchie, J Lewis): 219–62. Sage Publications, 2003.

[16] Stewart-Brown S, Janmohamed K. *Warwick-Edinburgh Mental Well-being Scale. User Guide Version 1* NHS Health Scotland, 2008 (http://www.mentalhealthpromotion.net/resources/user-guide.pdf).

[17] Ogundele MO, Omenaka IL. An audit of transitional care for adolescents with ADHD in a North West England district. Arch Dis Child 2012; 97 (suppl 1): A129-A.

[18] National Institute for Health and Care Excellence. NG87 Attention Deficit Hyperactivity Disorder: Diagnosis and Management. NICE, 2018. 29634174

[19] Kirby AV, Baranek GT, Fox L. Longitudinal predictors of outcomes for adults with autism spectrum disorder systematic review. OTJR: Occupation. Participation Health 2016; 36: 55–64. 10.1177/1539449216650182 27504878

[20] Broad KL, Sandhu VK, Sunderji N, Charach A. Youth experiences of transition from child mental health services to adult mental health services: a qualitative thematic synthesis. BMC Psychiatry 2017; 17: 380. 29183289 10.1186/s12888-017-1538-1 PMC5706294

[21] Sedgewick F, Hill V, Pellicano E. ‘It's different for girls’: gender differences in the friendships and conflict of autistic and neurotypical adolescents. Autism 2019; 23: 1119–32. 30280923 10.1177/1362361318794930

[22] Cribb S, Kenny L, Pellicano E. ‘I definitely feel more in control of my life’: the perspectives of young autistic people and their parents on emerging adulthood. Autism 2019; 23: 1765–81. 30818981 10.1177/1362361319830029

[23] Tatlow-Golden M, Gavin B, McNamara N, Singh S, Ford T, Paul M, Transitioning from child and adolescent mental health services with attention-deficit hyperactivity disorder in Ireland: case note review. Early Interv Psychiatry 2018; 12: 505–12. 28488369 10.1111/eip.12408

[24] Turgay A, Goodman D, Asherson P, Lasser R, Babcock T, Pucci M, Group ATPMW. Lifespan persistence of ADHD: the life transition model and its application. J Clin Psychiatry 2012; 73: 192–201. 22313720 10.4088/JCP.10m06628

[25] Berney T. Services for adults with autism spectrum disorders. Adv Mental Health Learning Disabil 2007; 1: 45–7.

[26] Lake JK, Perry A, Lunsky Y. Mental health services for individuals with high functioning autism spectrum disorder. Autism Res Treat 2014; 2014: 502420. 25276425 10.1155/2014/502420 PMC4168143

[27] Department of Health. Think Autism. Fulfilling and Rewarding Lives, the Strategy for Adults with Autism in England: An Update. Department of Health, 2014.

[28] Southby K, Robinson O. Information, advocacy and signposting as a low-level support for adults with high-functioning autism spectrum disorder: an example from the UK. J Autism Dev Disord 2018; 48: 511–9. 29063482 10.1007/s10803-017-3331-x PMC5807481

[29] Snell-Rood C, Ruble L, Kleinert H, McGrew JH, Adams M, Rodgers A, Stakeholder perspectives on transition planning, implementation, and outcomes for students with autism spectrum disorder. Autism [Epub ahead of print] 20 Jan 2020 Available from: 10.1177/1362361319894827. PMC7311242 31957461

